# Multiplane and Spectrally-Resolved Single Molecule Localization Microscopy with Industrial Grade CMOS cameras

**DOI:** 10.1038/s41598-018-19981-z

**Published:** 2018-01-29

**Authors:** Hazen P. Babcock

**Affiliations:** Center for Advanced Imaging, Harvard University, Cambridge, MA 02138 USA

## Abstract

This work explores the use of industrial grade CMOS cameras for single molecule localization microscopy (SMLM). We show that industrial grade CMOS cameras approach the performance of scientific grade CMOS cameras at a fraction of the cost. This makes it more economically feasible to construct high-performance imaging systems with multiple cameras that are capable of a diversity of applications. In particular we demonstrate the use of industrial CMOS cameras for biplane, multiplane and spectrally resolved SMLM. We also provide open-source software for simultaneous control of multiple CMOS cameras and for the reduction of the movies that are acquired to super-resolution images.

## Introduction

Super resolution imaging by localizing single fluorescent molecules is popular due to its comparative simplicity. This approach requires the detection of the signals from single fluorescent dye molecules. This signal is typically of order 10–1000 photons per pixel at the magnifications used in most SMLM microscopy setups. Thus the ideal camera should have a high quantum efficiency (QE) for optimal conversion of photons to photo-electrons (e-). Furthermore, as the signal is Poisson distributed the camera readout noise should be significantly less than $\sqrt{Ne-}$ in order to minimally contribute to the total noise. Due to these requirements it is generally accepted that SMLM requires the use of high end scientific cameras.

In two of the three initial demonstrations of the SMLM approach^1–3^ and much of the subsequent work EMCCDs have been the detector of choice. These cameras have a maximum QE of over 80% and the EM gain stage amplifies the signal such that the relatively large (20e- to 40e-) readout noise of the CCD does not dominate the Poisson noise of the signal. More recently scientific CMOS (sCMOS) cameras have become popular due to their much higher readout rates, greater pixel counts and reduced cost^4–7^. Modern sCMOS cameras also have a maximum QE of over 80% and readout noises of ∼1e-, which is small in comparison to the Poisson noise of the signal. Interestingly, high end industrial grade CMOS cameras are rapidly approaching the performance of sCMOS cameras. In particular it is not hard to find industrial CMOS cameras with 70% maximum QE and read noises of ∼2e- for $1.5 k, an order of magnitude less than the approximately $20 k cost of a typical sCMOS camera. This level of performance is sufficient for most types of SMLM imaging and there is little measurable difference in the quality of the final SMLM image from these cameras as compared to sCMOS cameras^8^.

This work follows earlier work that demonstrated several approaches to reducing the overall cost of a SMLM setup^8–10^. However here we take advantage of the reduction in camera cost first described in Ma *et al*.^8^ and use it to build a relatively inexpensive 4 camera setup. This is advantageous as it is simpler to construct a multiplane and/or multicolor imaging setup using a single camera per focal plane or color than it is to combine the focal planes or colors onto a single camera. In addition the final field of view of such a setup can be significantly larger as sharing the active area of a single camera is no longer necessary. Our setup can be quickly re-configured for different applications simply by swapping dichroics and/or beam splitters and adjusting lens positions. We demonstrate the use of this setup to acquire 2D SMLM images with a single camera, biplane and quadplane 3D SMLM images with 2 and 4 cameras respectively, and spectrally resolved SMLM (SR-STORM^11^) images with 4 cameras.

## Results

**Figure 1:**
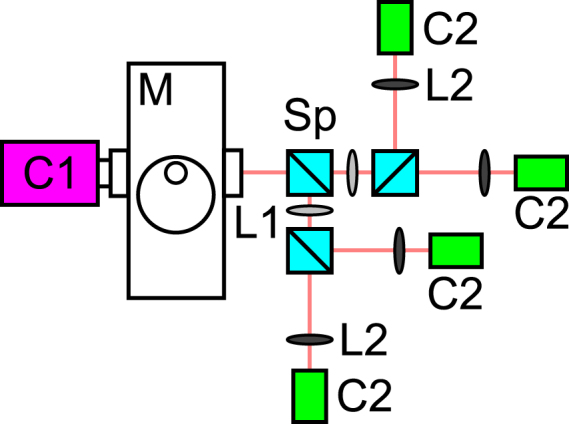
Schematic of the setup used for camera testing. **C1** (magenta) is a Hamamatsu ORCA-Flash4.0 camera, **M** is a Nikon TiU microscope, **Sp** (light blue) are fluorescent filter cube holders, **L1** (light gray) *f* = 125 mm lenses, **L2** (dark gray) *f* = 60 mm lenses and **C2** (green) are FLIR Imaging GS3-U3-51S5M-C cameras.

To quantitatively measure the difference in performance between industrial and scientific grade CMOS cameras we built the setup shown in Fig. 1. The setup is constructed around an inverted microscope (TiU, Nikon) mounted on an optical table (RS2000, Newport). This microscope has an optical port selector that allows one to quickly change which camera the sample is imaged onto. A single sCMOS camera (ORCA-Flash4.0 v2, Hamamatsu Photonics) was mounted directly onto the left port of the microscope. An optical cage system was used to mount 4 industrial CMOS cameras (GS3-U3-51S5M-C, FLIR Imaging) onto the right port of the microscope. The setup can be configured to use from 1 to 4 of the CMOS cameras at once by adding or removing dichroic beam splitters in the fluorescent filter cube holders (**Sp** in Fig. 1, DFM1, Thorlabs). In order to adjust for the difference in pixel size between the two cameras (ORCA-Flash4.0–6.5 μm, GS3-U3-51S5M-C - 3.45 μm) a 2x demagnifying lens pair was used (2*f* lens pair **L1**, **L2** in Fig. 1). A 60x 1.4NA oil immersion objective (CFI Plan Apo Lambda 60X , Nikon) was used for imaging, giving final pixel sizes of 108 nm for the ORCA-Flash4.0 camera and 120 nm for the GS3-U3-51S5M-C cameras.

We first tested how well the two cameras could localize 0.1 mm 580/605 fluorescent beads (F8801, Molecular Probes). The experiment was a side-by-side comparison of the relative performance of the CMOS and sCMOS cameras imaging exactly the same beads. Samples were prepared by sparsely and non-specifically immobilizing the beads onto a microscope coverslip. All of the fluorescence was imaged onto a single CMOS camera by removing the dichroics from the optical cage system. Pairs of 100 frame movies of the same field of view were taken at 100Hz with the sCMOS and CMOS cameras. The photon flux from the beads was adjusted to cover the range that is expected from single fluorescent dye molecules during SMLM imaging. The photo-bleaching of the beads during the acquisition of the two movies was negligible as these beads are very bright and require very little illumination laser power under these conditions. Analysis of the data was done by first finding the affine transform that best overlaid the images from the two different cameras. Then beads were identified and localized in movies from the sCMOS camera using a Python/C open-source implementation^12^ of the sCMOS analysis algorithm described in Huang *et al*.^5^. Next the bead fit locations were mapped to the CMOS camera and used as the starting points for fitting using the same sCMOS analysis algorithm. Beads were tracked through the movies by assigning all the beads that localized to within 2 pixel of a bead position in the first frame to the same track. Tracks where beads were missing in more than 10% of the frames in data from either of the cameras were discarded. The average intensity per bead and the average background used in the Cramer-Rao bounds calculations described below were determined using the values returned by the fitting algorithm.

**Figure 2:**
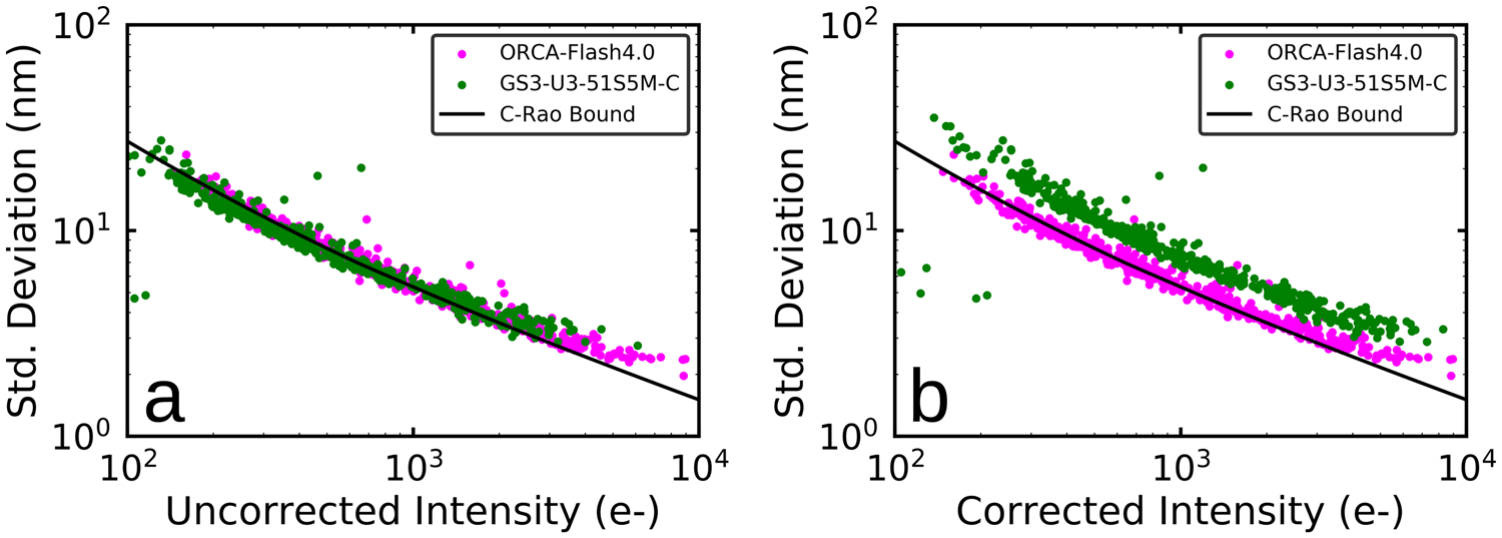
Comparison of localization precision measured using fluorescent beads (log-log plot). (**a**) Localization precision versus bead fluorescence intensity as measured by each camera. (**b**) Localization precision versus bead fluorescence intensity with the intensity reported by the GS3-U3-51S5M-C camera scaled by this camera’s sensitivity relative to the ORCA-Flash4.0 camera. The black line in both figures is the Cramer-Rao bound on the maximum localization precision.

Both cameras achieve the same localization precision as a function of bead intensity (Fig. 2a). This shows that the additional readout noise of the CMOS camera (1.6e- median at 100 Hz) as compared to the sCMOS camera (0.9e- median at 100 Hz) has little effect on the localization precision even at the lowest intensities tested. The sCMOS camera however has a higher QE than the CMOS camera, so it will detect more photons per bead than the CMOS camera, and will demonstrate superior localization precision once this is corrected for. We measured the difference in the number of photo-electrons detected per bead between the two cameras by plotting the bead intensity measured by the sCMOS camera versus that measured by the CMOS camera for each pair of beads and fitting a line. We found that the CMOS camera detected on average ∼50% fewer photo-electrons. Figure 2b is a plot of the localization precision with the intensity values from the CMOS camera corrected for this difference in detection efficiency. As expected, the localization precision of the sCMOS camera is now about 40% better. It is also worth mentioning that this CMOS camera should perform as well as an EMCCD camera in SMLM applications with reasonably bright dyes (Supplementary Figs S3, S4). The excess noise added in the EMCCD gain stage is equivalent to reducing the signal by a factor of 2, so the performance of a perfect EMCCD would be approximately that of a CMOS camera with 50% QE^13^. Figure 2 also shows that the localization precision from both cameras, without correcting for the difference in detection efficiency, achieves the Cramer-Rao theoretical bound. The Cramer-Rao theoretical bound was calculated by numerical integration of equation 5 in Mortensen *et al*.^13^. A pixel size of 108 nm and a PSF *σ* of 130 nm were used as the constants in this equation. The PSF *σ* value was the average value returned by the sCMOS analysis algorithm for the width of the Gaussian fit to each localization. The source of the apparent deviation at the highest intensities is uncertain, but was also observed in other recent work^9^.

The suitability of the CMOS camera for 2D SMLM imaging was next evaluated with a sample consisting of microtubules in U-2 OS cells labeled with the Alexa-647 fluorescent dye. In this experiment we took advantage of the fact that a single Alexa-647 dye molecule can be localized multiple times in order to acquire SMLM images of the same field of view with both the sCMOS and the CMOS cameras. In order to minimize the relative effects of photo-bleaching on the the final SMLM image we alternated imaging between the two cameras, acquiring relatively short movies with each camera before switching to the other camera. The procedure that was followed was an initial turn off phase followed by a 5 k frame SMLM movie with the sCMOS camera. Then alternating 10k frame SMLM movies were taken with the CMOS camera and the sCMOS camera, and finally a 5 k frame SMLM movie was taken with the sCMOS camera. The total SMLM movie length for each camera was 70 k frames. All movies were taken at a frame rate of 100 Hz with HILO illumination^14^. An affine transform was used to convert the CMOS localization positions to the sCMOS coordinate system.

**Figure 3:**
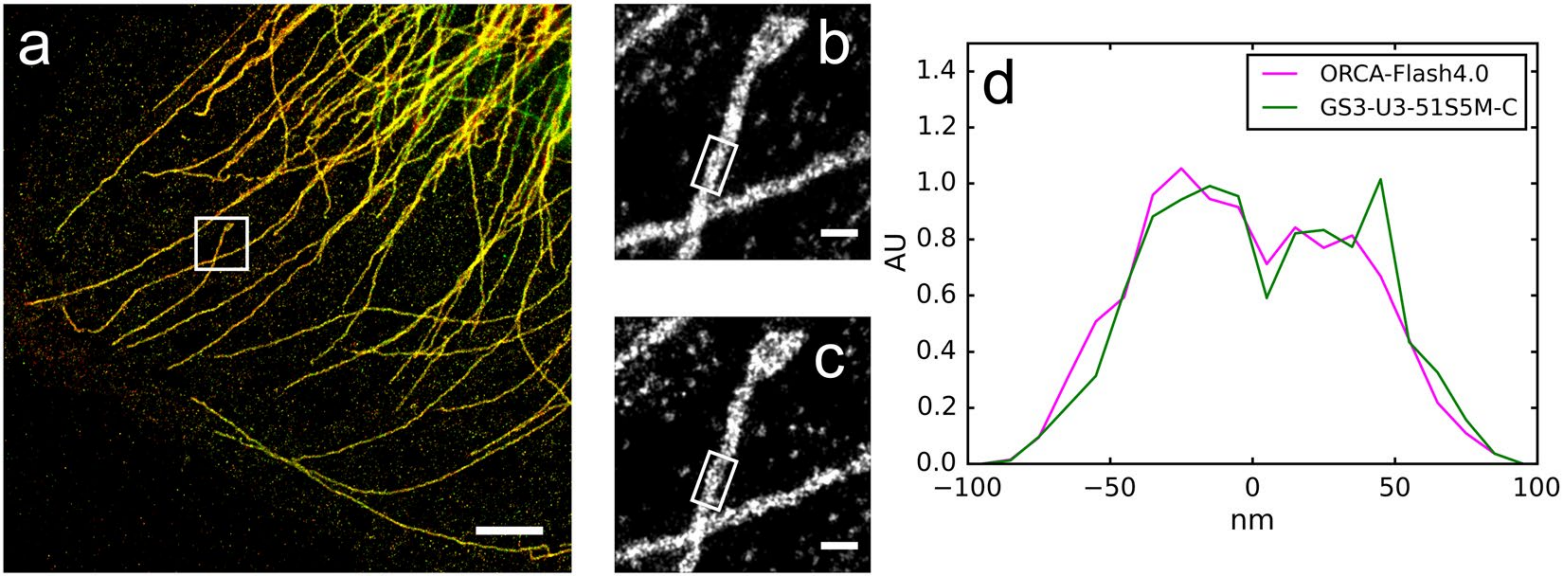
Comparison of 2D SMLM images of Alexa-647 labeled microtubules in U2OS cells acquired with an ORCA-Flash4.0 camera and a GS3-U3-51S5M-C camera. (**a**) Overlay of the two images (red - ORCA-Flash4.0, green - GS3-U3-51S5M-C). (**b**) Zoom in of the area white boxed area in (**a**), ORCA-Flash4.0 camera. (**c**) Zoom in of the white boxed area in (**a**), GS3-U3-51S5M-C camera. (**d**) Histograms of the white boxed areas in (**b**,**c**) for the two cameras. Scale bars are 2 μm in (**a**), 200 nm in (**b**,**c**).

There was little visible difference in the SMLM images from the CMOS and the sCMOS cameras, as shown in Fig. 3. All of the microtubules in Fig. 3a are yellow indicating excellent agreement between the sCMOS image (red) and the CMOS image (green). The higher resolution zoomed images in Fig. 3b,c from the two cameras are also identical. Finally, both cameras were able to resolve the hollow structure of the immunostained microtubules in some areas of the image (Fig. 3d). The sCMOS camera identified 8.8 M localizations and the CMOS camera identified 6.8 M localizations. However the localizations detected by the sCMOS camera were significantly brighter with an average intensity of 9100e- (53e- background) compared to 5200e- (39e- background) for the CMOS camera. Using these values and the Cramer-Rao bound formula in^13^ gives a theoretical resolution of *σ* = 1.7 nm for the sCMOS camera and *σ* = 2.3 nm for the CMOS camera. As the real resolution is additionally effected by issues such as increased error from fits of overlapping emitters and imperfect drift correction the small difference in the localization precision between the two cameras is very difficult to detect. The SMLM sub-image cross-correlation approach was used for drift correction in all the SMLM images in this paper.

Having established that this CMOS camera works for single imaging plane SMLM we next used it for biplane 3D SMLM microscopy^15–17^. This is a more stringent test of camera performance as in this geometry the fluorescence from a single dye is split by a 50/50 dichroic beam splitter, with half of the light going to each CMOS camera. Lenses **L2** in Fig. 1 were adjusted so that the focal planes of the two cameras were offset by ∼600 nm. An affine transform was used to map X,Y positions from the second camera to the first camera. The z dependence of the PSF shape for both cameras was measured by scanning 0.1 μm beads fixed to a coverslip through the focal planes of the two cameras using a piezo objective positioner. Image stacks of the average PSF as a function of z were created only for beads that were visible on both cameras, and that were at least 24 pixels from neighboring beads. A cubic spline was fit to the average PSF image stacks in order to model the shape of the PSF as a function of z for each camera^18^. The cubic splines were used for analysis of biplane SMLM movies with the cubic spline fitting algorithm described in^18^ adapted for multiplane sCMOS analysis and available here^12^. This algorithm uses the SNSMIL algorithm described in Tang *et al*.^19^ for localization identification.

**Figure 4:**
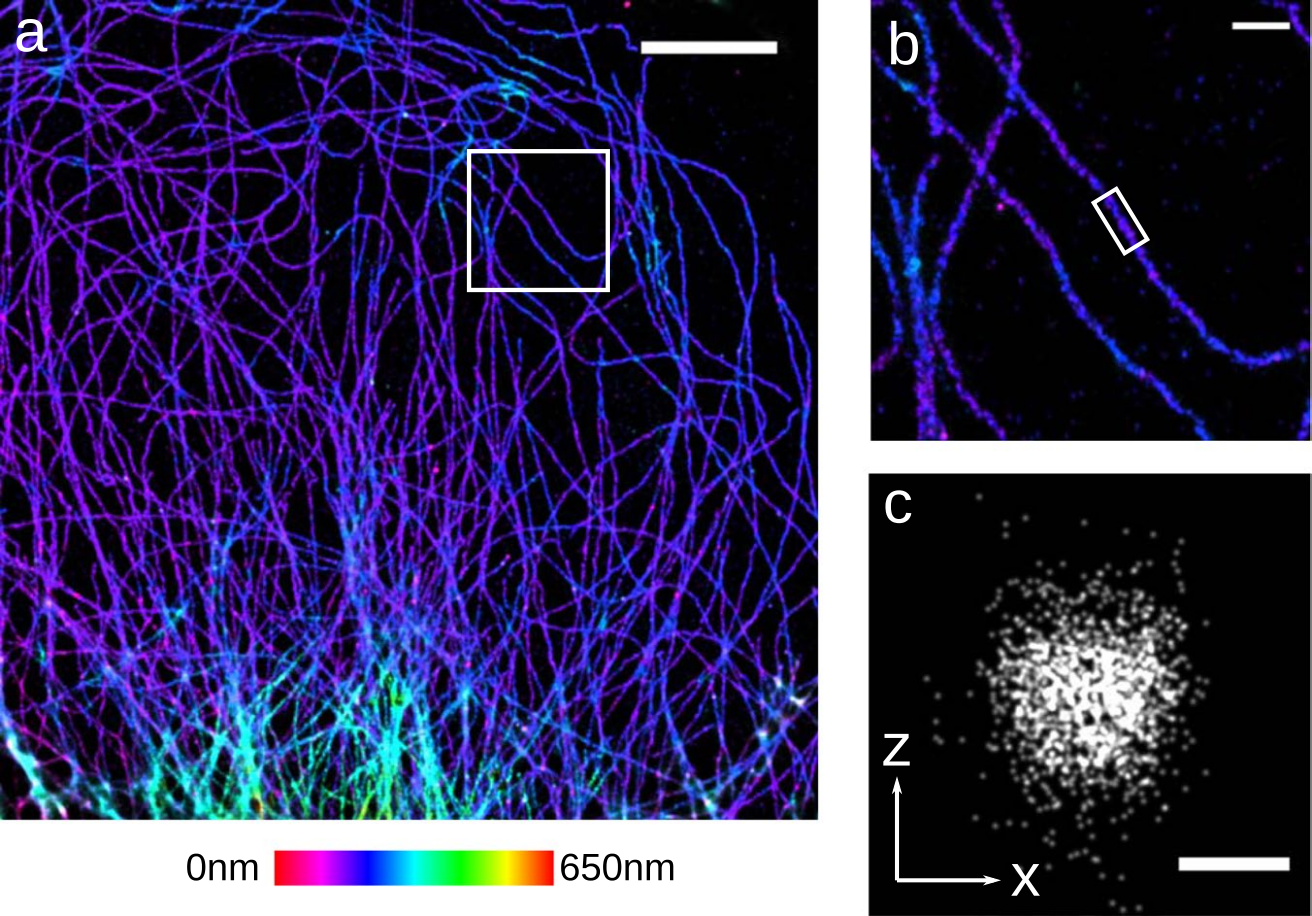
3D SMLM image of Alexa-647 labeled microtubules in U2OS cells taken using two GS3-U3-51S5M-C cameras in a biplane configuration. (**a**) 3D SMLM image with Z color scale as shown in the color bar. (**b**) Zoom in of the area white boxed area in (**a**) with the same Z color scale. (**c**) An X/Z cross section of the microtubule in the white boxed area in (**b**). Gaussian fits of the width of microtubule in (**c**) give *σ* = 23.6 nm in X,Y and 22.3 nm in Z. Scale bars are 5 μm in (**a**), 500 nm in (**b**), 50 nm in (**c**).

We were able to acquire biplane 3D SMLM images with this CMOS camera (Fig. 4). The sample again was microtubules in fixed U-2 OS cells labeled with the Alexa-647 fluorescent dye. The Alexa-647 dye is one of the brightest SMLM compatible dyes and is expected to emit ∼6000 photons on average per switching cycle in our imaging conditions^20^. It may be more difficult to acquire biplane SMLM images of dimmer photo-switchable proteins using this CMOS camera. The localizations that were identified using SNSMIL threshold of 8 *σ* had an average intensity of 3640 e- and a background of 30e-/plane. Using these values and the Cramer-Rao formalism in^21^ the theoretically estimated resolution is *σ* = 5.6 nm in X,Y and *σ* = 21.1 nm in Z at a z value that is halfway between the two camera planes. The actual X,Y resolution is however likely at least 2x worse than these values as the hollow structure of the immunostained microtubules is no longer resolvable (Fig. 4c). In this and subsequent figures the z value returned by the fitter was multiplied by 0.79 as a first order correction for spherical aberration^22,23^.

**Figure 5:**
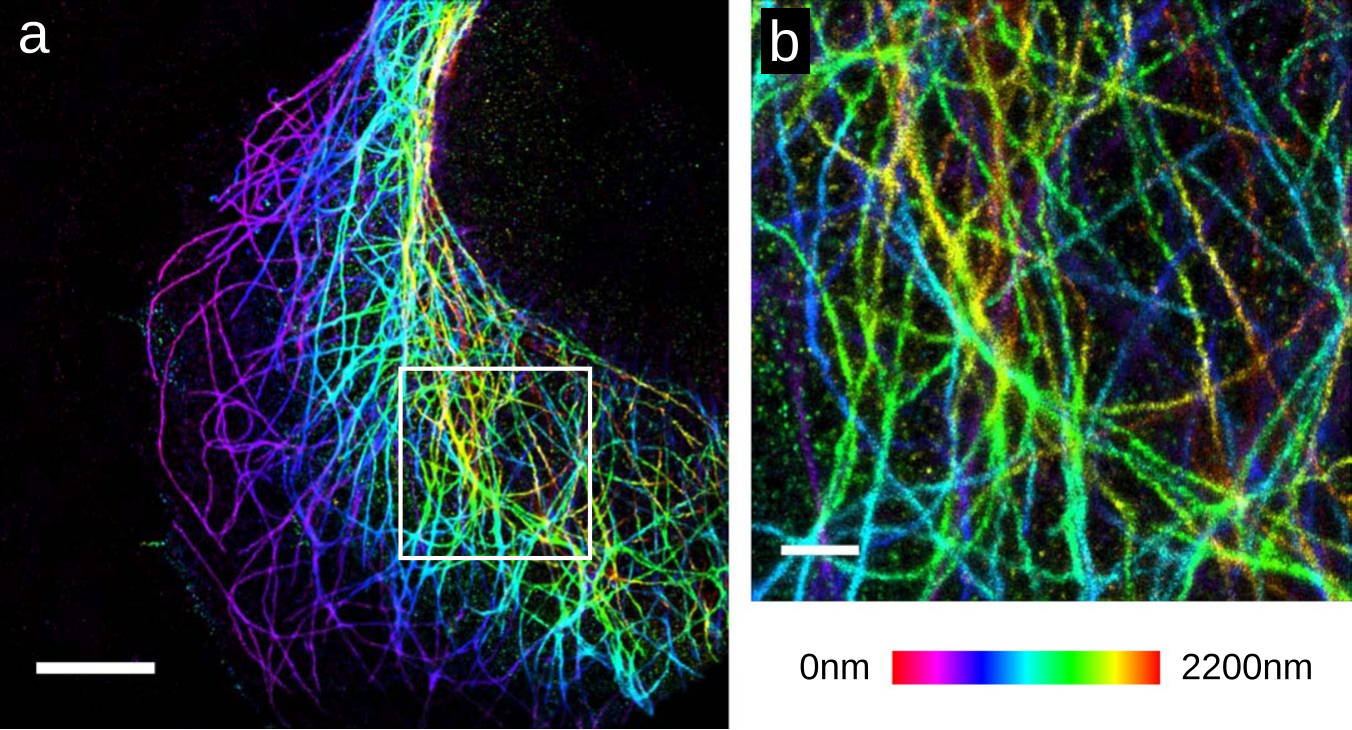
3D SMLM image of Alexa-647 labeled microtubules in U2OS cells taken using four GS3-U3-51S5M-C cameras in a quadplane configuration. (**a**) 3D SMLM image with Z color scale as shown in the color bar. (**b**) Zoom in of the area white boxed area in (**a**) with the same Z color scale. Scale bars are 5 μm in (**a**), 1 μm in (**b**).

Next we demonstrate the use of this CMOS camera for quadplane 3D SMLM microscopy. In the quadplane geometry the fluorescence from a single dye is split by 3 50/50 dichroic beam splitters onto 4 different cameras^24,25^, with each camera receiving 1/4 of the total signal. The imaging planes of the cameras were configured to be separated by ∼500 nm enabling the simultaneous acquisition of an ∼2.0 μm thick image slice. We followed the same procedure as for biplane imaging, an affine transform was used to map X,Y positions from the second, third and fourth cameras to the first camera. Cubic splines modeling the PSF for each camera were constructed from z scan image stacks of 0.1 μm beads. The cubic splines were used by the multiplane sCMOS cubic spline algorithm to localize single fluorescent dye molecules^12,18^. A quadplane SMLM image of microtubules labeled with the Alexa-647 fluorescent dye is shown in Fig. 5. The localizations that were identified using a SNSMIL threshold of 6 *σ* had an average intensity of 4940e- and a background of 35.5e-/plane. Using these values the theoretically estimated resolution is *σ* = 8.3 nm in X,Y and *σ* = 19.4 nm in Z, though as above, it is unlikely that the actual resolution is this high.

Finally we demonstrate the use of this CMOS camera for a new approach to SR-STORM^11^. In SR-STORM dyes with emission wavelength differences of order 20 nm are distinguished by simultaneously measuring a localizations spectrum and position. This allows one to do multicolor imaging with dyes that are similar to the Alexa-647 dye. This is an advantage as Alexa-647 is currently one of the best dyes for SMLM imaging, and these dyes all have similar performance to Alexa-647. In our approach a series of long-pass dichroics was arranged to create 4 different color channels with one camera per color channel, instead of using a prism to spectrally resolve different dye molecules. One advantage of this approach is that it tolerates moderately higher localization densities as the dye spectrum is not spread across as many camera pixels. Another advantage of this approach is that all of the fluorescence is used for both localization and color determination. In contrast, in the original SR-STORM approach half of the fluorescence is used to measure the spectrum and the other half is used to measure localization position. A disadvantage of this approach is that the ability to precisely measure the spectrum of a localization is lost. Localization color is instead identified by the relative signal received in each color channel. We note that with this approach it is also possible to simultaneously determine the z position of the localization. The 4 cameras each had relatively small ∼100 nm offsets between their focal planes which allowed us to determine z using the same approach as for quadplane imaging. The total z range in this geometry is however limited to about 500 nm as localizations need to be roughly in focus on all 4 cameras simultaneously in order to accurately measure the relative signal in each color channel.

We first imaged dye labeled secondary antibodies non-specifically bound to coverslips. The dyes we choose were DyLight-633, Alexa-647, CF660C and CF680, all of which work well for SMLM imaging as they are bright in their on state, have a duty cycle of ∼1:1000 and switch multiple times^11,20^. To measure our ability to discriminate between these dyes, each dye was imaged as a separate sample, then localizations of known dye type were merged into a single data set. We found that by simply computing the first moment of the signal as a function of color channel we could distinguish the dyes with a maximum cross-talk of 5% (DyLight-633 and Alexa-647)(Supplementary Fig. S5). The maximum cross-talk could be reduced to 2% using a clustering approach. A 4 component vector was created for each localization from the relative signal in each color channel. Then k-means clustering was used to partition the localizations into clusters based on their 4-vectors. Finally, those localizations whose 4-vectors were in the top 20% in terms of distance from the nearest cluster mean were discarded.

**Figure 6:**
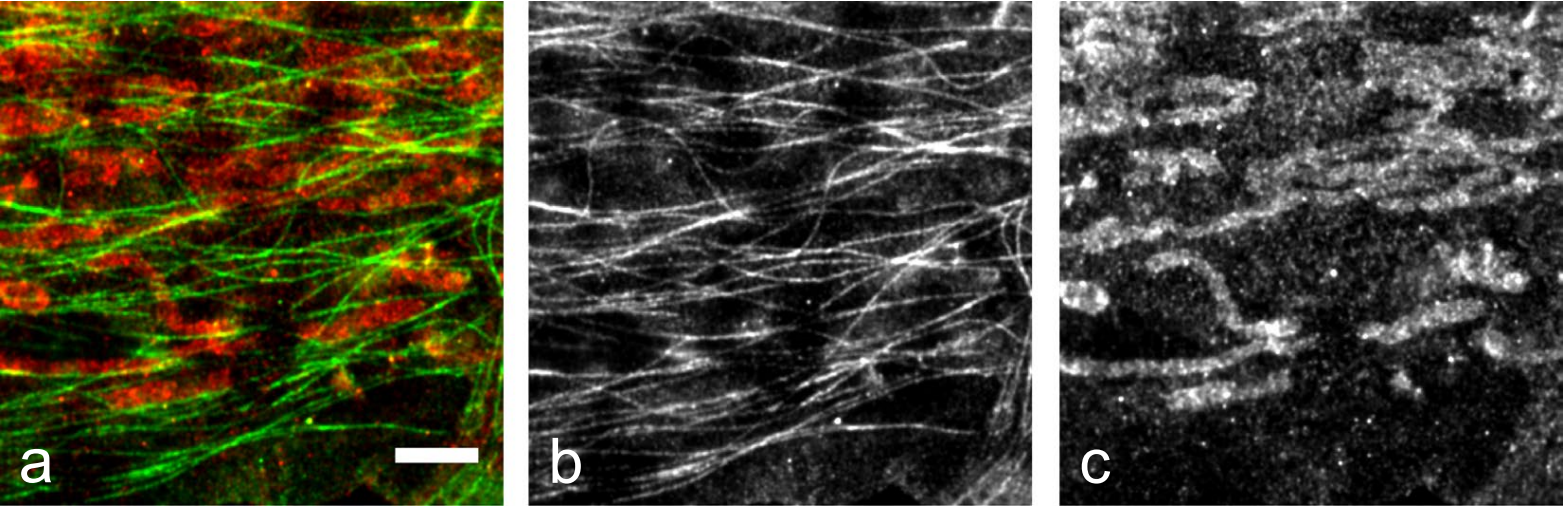
SR-STORM SMLM image of Alexa-647 labeled microtubules and Biotium CF680 labeled mitochondria in U2OS cells. The data was acquired using four GS3-U3-51S5M-C cameras, each detecting a different color channel. (**a**) Two color image with microtubules shown in green and mitochondria shown in red. (**b**) Gray-scale image showing the localizations assigned to the Alexa-647 category (microtubule). (**c**) Gray-scale image showing the localizations assigned to the CF680 category (mitochondria). The scale bar is 2 μm.

A SR-STORM image of microtubules and mitochondria is shown in Fig. 6. In this image microtubules were labeled with the Alexa-647 fluorescent dye and the mitochondrial protein TOMM20 was labeled with the Biotium CF680 dye. The localizations were categorized as Alexa-647 or CF680 based on the distance between their 4-vector and cluster mean vectors. The cluster mean vectors were determined by k-means clustering of an Alexa-674 and a CF680 data set acquired as described in the previous paragraph. We discarded those localizations whose 4-vectors were in the top 20% in terms of distance from the nearest cluster mean.

We also provide measurements of the X, Y and Z resolutions of the above approaches as function of localization brightness in supplementary Fig. S6. As above, the resolution was characterized using 100 frame movies of 0.1 μm 580/605 nm fluorescent beads acquired at 100 Hz. These measurements are representative of the best case resolution as there is very little background fluorescence or sample drift and there are no overlapping emitters. As expected, they show that quadplane imaging requires on average ∼2x more photons to achieve the same resolution as biplane imaging because at the z position tested only two focal planes make significant contributions to the localization measurement. The measurement also shows that the X,Y resolution in SR-STORM is better than biplane or quadplane, but the Z resolution is worse. This is likely due the relatively small offset in the focal positions of the 4 different planes, which improves X,Y resolution at the expense of Z resolution.

## Discussion

In this work we evaluated the use of high end industrial CMOS cameras for SMLM microscopy. We found that they are well suited for many SMLM applications. However they are not yet as sensitive as the best sCMOS cameras so they may not be a good choice for experiments involving dimmer dyes. The more than an order of magnitude reduction in camera cost makes it reasonable to consider building microscopy setups that have 4, 8 or possibly even more cameras. This could enable extremely high throughput imaging as well as novel imaging approaches. For example, an octa-plane setup with a ∼750 nm spacing between each camera could be used to acquire a z-stack image of an entire cell or thin tissue section in a single exposure. Octa-plane SMLM imaging could be possible with very bright dyes^26^ or approaches like DNA-PAINT^27^. These cameras could also be used for multiplane structured illumination microscopy^28^ or standard epi-fluorescence microscopy in combination with the algorithms to remove sCMOS noise from the final images^29^.

## Methods

### Acquisition Hardware and Software

Other important components of the setup that are not shown in Fig. 1 include 1 W 560 nm and 1 W 647 nm fiber lasers (2RU-VFL-P-1000-560-B1R, 2RU-VFL-P-1000-647-B1R, MPB photonics) used for fluorescence illumination. The lasers provided even illumination across a 40 μm diameter field of view, with typical powers for SMLM imaging of 3–6 KW/cm^2^ at the sample. A custom multi-band dichroic (zt405/488/561/647/752rpc, Chroma) and a custom multi-band emission filter (zet405/488/561/647-656/75, Chroma) were used for all of the experiments except SR-STORM. A 652 dichroic (FF652-DI01-25X 36, AVRO Inc.) and a 647 long pass emission filter (LP02-647RU-25, AVRO Inc.) were used for the SR-STORM experiments. 50/50 dichroic beam splitters (BSW10R, Thorlabs) were used to split the fluorescence emission between different CMOS cameras in the biplane and quadplane experiments. 670 nm, 695 nm and 720 nm dichroic filters (T670lpxr, T695lpxr and 720dcxr, Chroma) were used to spectrally separate the fluorescence emission in the SR-STORM experiments. A 980 nm IR laser diode (LP980-SF15, Thorlabs), USB camera (DCC1645C, Thorlabs) and a piezo objective positioner (Nano-F100S, Mad City Labs) were used to build a IR reflectance based focus lock system that corrected for focal drift during data acquisition^23^.

The setup was controlled with custom software written in Python3 and that used the PyQt5 GUI library. This software was designed to control multiple cameras at once even if they have different chip sizes and/or are from different manufacturers. It currently supports CMOS cameras from Andor, Hamamatsu and FLIR Imaging. The number and type of cameras that are controlled as well as additional setup functionality is specified in a single XML file in order to make it more straightforward to use the software to control different setups. This software is open-source and is available on Github^30^.

The CMOS cameras have a sensor that is 2448 × 2048 pixels and which operates in global shutter mode. The CMOS cameras were controlled using the USB3 interface and a dedicated USB3 PCI Express card (PEXUSB3S7, Startech). In 12 bit video mode 7 we measured the following maximum frame rates for a single camera, 37 Hz at 2448 × 2048, 252 Hz at 512 × 512, 463 Hz 256 × 256 and 796 Hz at 128 × 128. The camera exposures were synchronized using their GPIO connectors for experiments in which more than one camera was used. The SMLM movies were streamed directly onto a 1TB PCIe M.2 solid state drive (SSD) (MZ-V6E1T0BW, Samsung).

### Immunofluorescence and Imaging

Microtubules in fixed U-2 OS cells were fluorescently labeled using the following protocol. Cells cultured in 8 well chambers with a #1.5 coverslip bottom were washed with phosphate buffered saline (PBS), permeabilized for 1 minute with a buffer containing 0.1 M Pipes, 0.2% triton X-100, 1 mM EGTA and 1 mM MgCl2, then fixed with a solution of 3% paraformaldehyde and 0.1% glutaraldehyde in PBS (PFA/GA) for 10 minutes. After fixation the cells were washed 3x with PBS then blocked for 15 minutes with 3% BSA, 0.1% triton X-100 in PBS (BB). The anti-beta tubulin primary antibody (ab6046, Abcam) was diluted to 2 μg/ul in BB and incubated for 30 minutes before removal by washing 3x with PBS. The goat anti-rabbit Alexa-647 labeled secondary antibody (A21245, Invitrogen) was diluted to 2 μg/ul in BB and incubated for 30 minutes before removal by washing 3x with PBS. The sample was then post-fixed for 10 minutes with PFA/GA, washed 3x with PBS and stored dry at 4 C until use.

Microtubules and mitochondria in fixed U-2 OS cells were fluorescently labeled using the same protocol as for microtubule labeling with the following modifications. The initial permeabilization step was removed, after the PBS wash the cells were immediately fixed with PFA/GA. Primary antibodies were rat anti-tubulin (ab6160, Abcam) and rabbit anti-TOMM20 (ab78547, Abcam) both at 2 μg/ul in BB. Secondary antibodies were Alexa-647 goat anti-rat (A21247, Life technologies) and CF680 donkey anti-rabbit (20820-50ul, Biotium) at 2 μg/ul in BB.

Imaging was performed in an oxygen scavenging buffer consisting of 100 mM Tris (pH 8.0), 50 mM NaCl, 0.5 mg/ml^−1^ glucose oxidase, 40 μg/ml^−1^ catalase, 10% glucose and 143 mM BME.

### Data Availability

Data in this paper is available by request.

## Electronic supplementary material

Supplementary Information
