# Supplementary material for: Multiplane and Spectrally-Resolved Single Molecule Localization Microscopy with Industrial Grade CMOS cameras
Source: Sci Rep. 2018 Jan 29;8:1726. doi: 10.1038/s41598-018-19981-z (PMC5789017; doi:10.1038/s41598-018-19981-z)
Supplement: Supplementary file 1 — Supplementary Information [file 41598_2018_19981_MOESM1_ESM.pdf]

Multiplane and Multicolor Single Molecule Localization Microscopy with Industrial Grade CMOS cameras

Hazen P. Babcock

Supplementary Figure S1 : sCMOS and CMOS Camera Pixel Defect Analysis

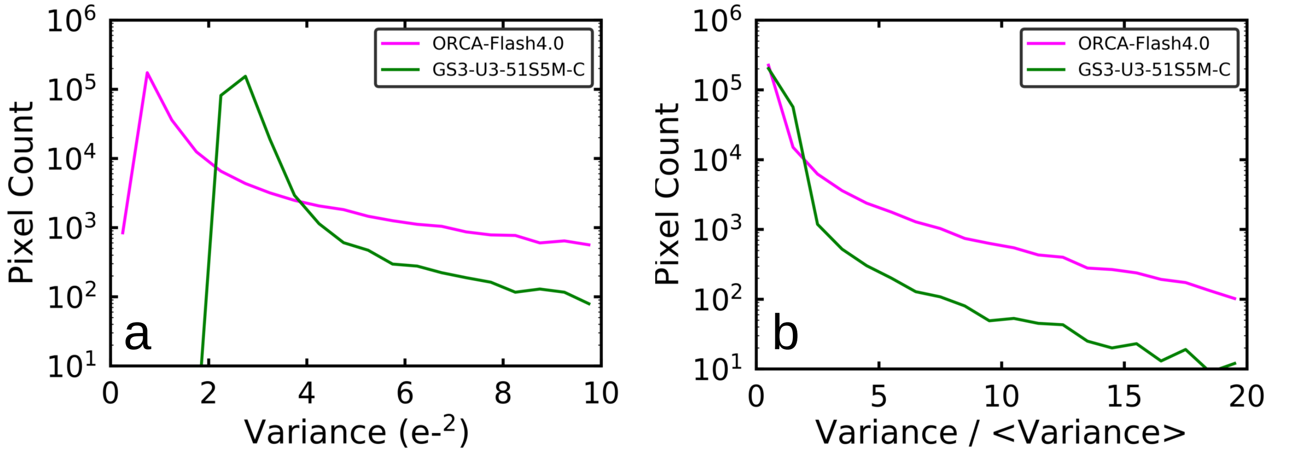

Figure S1. A comparison of the per pixel variance of a Hamamatsu ORCA Flash 4 v2 and a FLIR GS3-U3-51S5M-C camera. (a) A histogram of the measured variance for each pixel in units of e- (photo-electrons). (b) A histogram of the measured variance for each pixel normalized by the average variance for all pixels. The variance was measured for an ROI of 512 x 512 pixels centered on the detector chip. Data was acquired at a frame rate of 50 Hz. The median variance for the Hamamatsu ORCA Flash 4 v2 camera was 0.82e- and the median variance of the FLIR GS3-U3-51S5M-C was 2.6e-. These values are consistent with the manufacturers specifications. In addition we observed that 1.5% of the ORCA Flash 4 v2 camera pixels and 0.59% of the GS3-U3-51S5M-C camera pixels had variances that are 3σ larger than the mean variance.

Supplementary Figure S2 : sCMOS and CMOS Camera Response Linearity

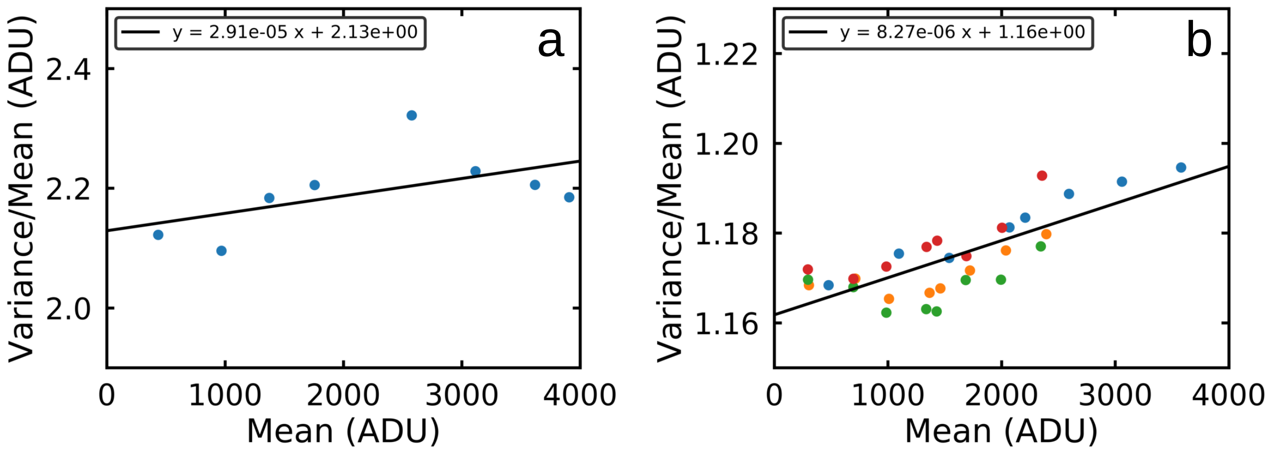

Figure S2. A measurement of the average response linearity of a Hamamatsu ORCA Flash 4 v2 (a) and 4 different FLIR GS3-US-51S5M-C cameras (b). The cameras were illuminated with an approximately uniform white light source at 8 different light intensities. The per pixel mean and variance of the central 512 x 512 pixels were calculated from 2000 frame long movies taken at 50 Hz. These values were then corrected for per pixel offsets and averaged together to create a single mean and variance value for each of the 8 intensities. The average variance divided by the average mean versus the average mean is plotted in (a) and (b) along with a best fit line. If the response of these cameras was linear we would expect the slope of the best fit line to be 0. The fact that it consistently has a small slope for all the cameras indicates that the response of these cameras is not quite linear, on average the per pixel gain increases slightly with increasing intensity.

Supplementary Figure S3 : Camera Performance Comparison Simulations

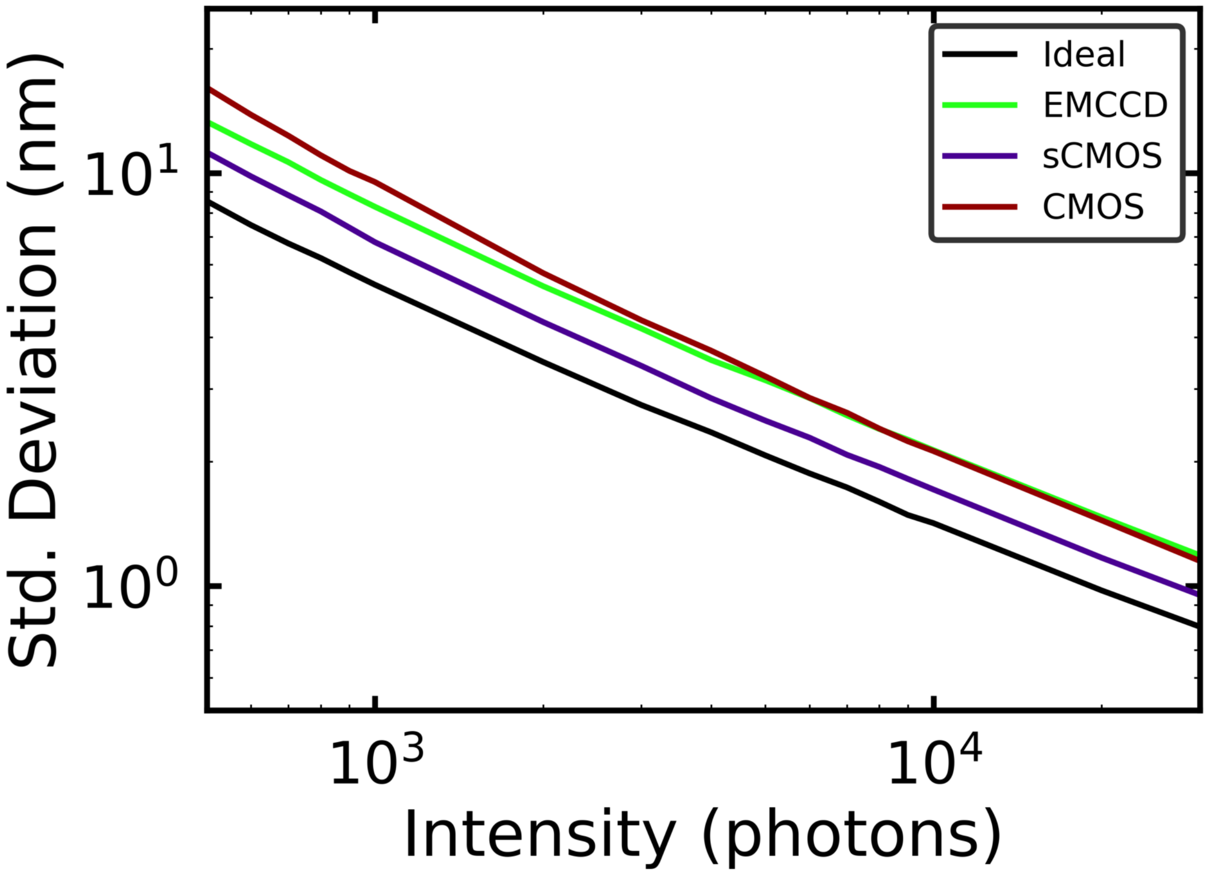

Figure S3. A comparison of the X,Y localization performance of 4 different cameras as determined from simulations. Simulated SMLM movies of isolated emitters as they would appear when imaged with 4 different cameras were generated using the simulator module in the storm-analysis Python project with a Gaussian PSF. The movies were then analyzed with either the 3D-DAOSTORM (Ideal and EMCCD) or the sCMOS (sCMOS and CMOS) modules in the storm-analysis project. The standard deviation of the difference between the localizations ground truth position and the measured position is plotted as a function of intensity. The parameters that were used for the different cameras are (1) Ideal - 100% QE, no readout noise, (2) EMCCD - 95% QE, 65e- readout noise, 30 EMCCD gain, 0.2 preamp gain, (3) sCMOS - 70% QE, 1e- readout noise and (4) CMOS - 50% QT, 2.4e- readout noise. These parameters were taken from the manufacturers documentation for an Andor Ultra 897 EMCCD operating at 10 MHz, a Hamamatsu ORCA Flash 4 v2 and a FLIR GS3-U3-51S5M-C all detecting 700 nm wavelength light. For each camera the number of photons was multiplied by the camera's QE to determine the number of photo-elections (e-) detected. A pixel size of 110 nm was used for each camera.

Supplementary Figure S4 : EMCCD versus CMOS Comparison

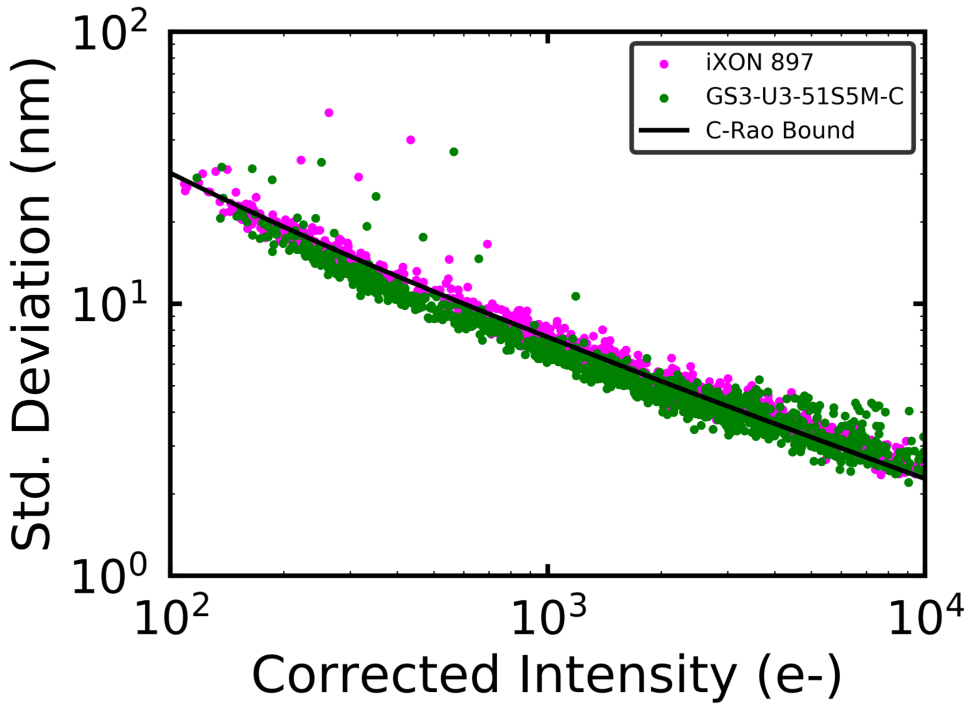

Figure S4. The localization accuracy of an Andor Ultra 897 EMCCD camera and a FLIR GS3-US-51S5M-C CMOS camera measured using 100 nm fluorescent beads. These measurements were performed in the same way as in the main text (Fig. 2). Briefly, 0.1 µm 580/605 nm fluorescent beads (F8801, Molecular Probes) were immobilized on a microscope coverslip. 100 frame movies of the exactly the same beads were acquired at 31 Hz by using the microscope's port selector to send the fluorescence emission to the desired camera. The image was also enlarged an additional 1.5x for the EMCCD camera using the microscope's built in 1x / 1.5x magnification selector. In these measurements the image pixel sizes were 177 nm for the EMCCD and 120 nm for the CMOS camera. We were not able to better match the image pixel sizes without additional modifications to the setup due to the large difference in physical pixel size between these two cameras, 16 µm for the EMCCD camera versus 3.45 µm for the CMOS camera. The Cramer-Rao bound for the EMCCD was calculated as described in the main text. A pixel size of 177 nm and a PSF σ of 130 nm were used as the constants in the Cramer-Rao calculation. The intensity values from the CMOS camera were corrected for a 65% difference in detection efficiency.

Supplementary Figure S5 : Color Channel Mean

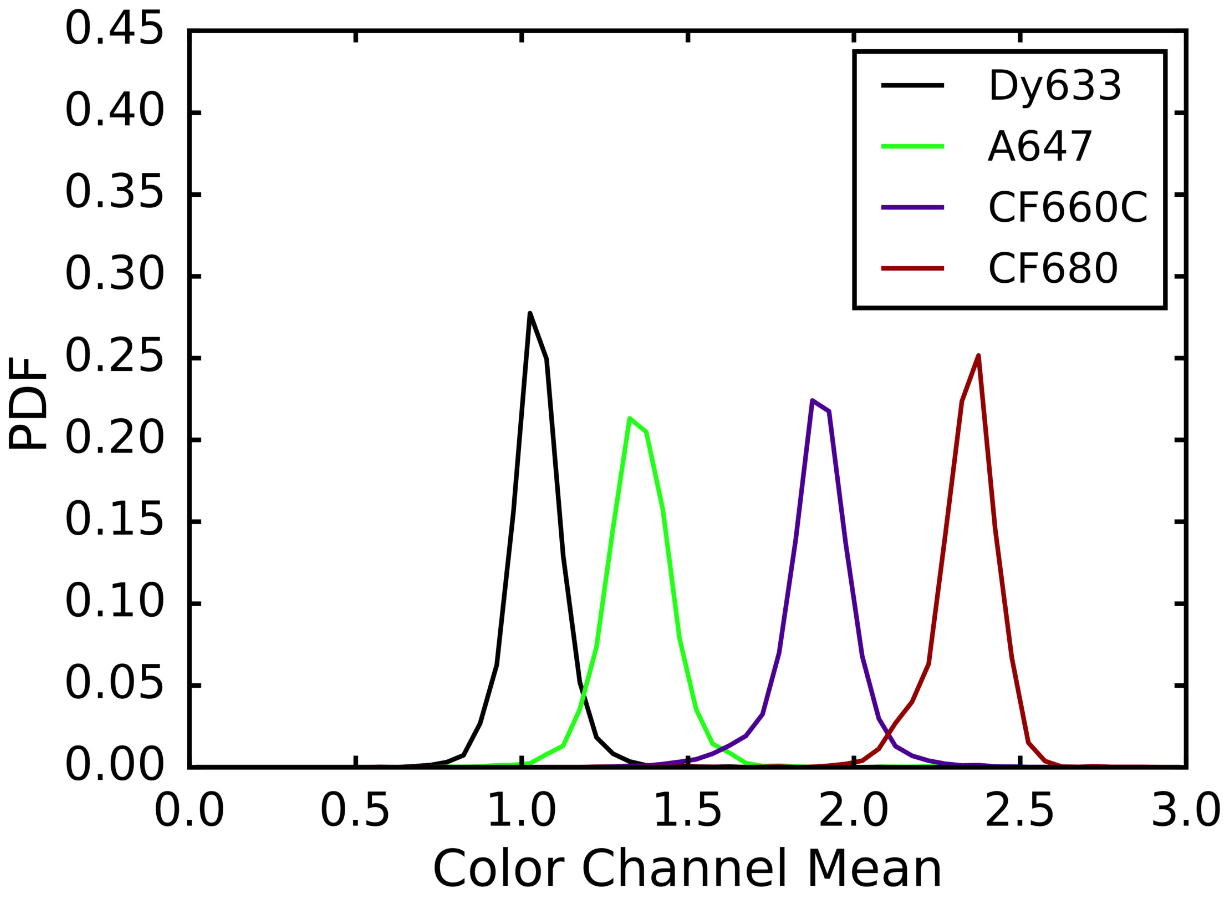

Figure S5. The probability distribution function of the dye color channel mean. Dye labeled secondary antibodies were diluted in PBS and non-specifically bound to coverslips. A single 500 frame STORM movie was taken of each type of dye and analyzed with the multiplane spline fitting software. For each localization the color channel mean statistic was calculated by multiplying the localizations height in the channel by the channel number and dividing by the total of the heights. This statistic is the first moment of the dye heights with respect to color channel number. The color channels were organized from shortest to longest wavelength with channels covering approximately the following wavelengths, channel0 647-670 nm, channel1 670-695 nm, channel2 695-720 nm, channel3 720+ nm . Localizations with a total height of less than 200 e- were removed from the analysis. Dye abbreviations are Dy633 for DyLight 633, A647 for Alexa 647, CF660C for Biotium CF660C and CF680 for Biotium CF680.

Supplementary Figure S6 : Biplane, Quadplane and SR-STORM X,Y,Z resolution

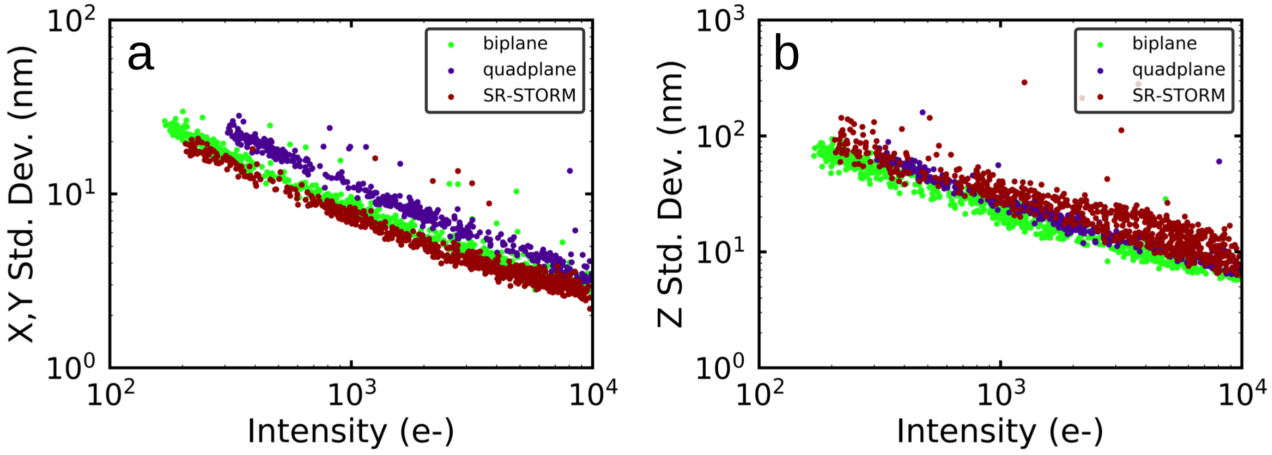

Figure S6. A comparison of X,Y (a) and Z (b) localization precision for the biplane, quadplane and SR-STORM imaging geometries. For each geometry multiple 100 frame movies of isolated 0.1 µm fluorescent beads were acquired at 100 Hz over a range of illumination intensities. All the movies for each geometry were taken at approximately the same z position. This z position was at the approximate center of the z range for biplane, quadplane and SR-STORM. It was half way between the two planes for biplane, half way between the middle two planes for quadplane and the position where the beads were best focused on all four cameras simultaneously in SR-STORM. Beads that were less than 17 pixels from their nearest neighbor or less than 10 pixels from the edge of the image were excluded from further analysis. The standard deviation of the bead position in x, y and z is plotted for those beads that were detected and localized in at least 95 frames. The intensity is the best fit value returned by the analysis summed across all of the planes.
